# Whole-body parametric mapping of tumour perfusion in metastatic prostate cancer using long axial field-of-view [^15^O]H_2_O PET

**DOI:** 10.1007/s00259-024-06799-3

**Published:** 2024-06-28

**Authors:** Mads Ryø Jochumsen, Nana L Christensen, Peter Iversen, Lars C Gormsen, Jens Sørensen, Lars P Tolbod

**Affiliations:** 1 Department of Nuclear Medicine and PET-Centre, Aarhus University Hospital, Palle Juul-Jensens Boulevard 165, Aarhus N, 8200 Denmark; 2 Department of Nuclear Medicine, Gødstrup Hospital, Herning, Denmark; 3 Department of Clinical Medicine, Aarhus University, Aarhus, Denmark; 4 Department of Surgical Sciences, Uppsala University Hospital, Uppsala, Sweden

**Keywords:** Tumour blood flow, Long axial field-of-view, Dynamic PET, Perfusion, Prostate cancer, Cancer

## Abstract

**Purpose:**

Tumour perfusion is a nutrient-agnostic biomarker for cancer metabolic rate. Use of tumour perfusion for cancer growth assessment has been limited by complicated image acquisition, image analysis and limited field-of-view scanners. Long axial field-of-view (LAFOV) PET scan using [^15^O]H_2_O, allows quantitative assessment of whole-body tumour perfusion. We created a tool for automated creation of quantitative parametric whole-body tumour perfusion images in metastatic cancer.

**Methods:**

Ten metastatic prostate cancer patients underwent dynamic LAFOV [^15^O]H_2_O PET (Siemens, Quadra) followed by [^18^F]PSMA-1007 PET. Perfusion was measured as [^15^O]H_2_O K_1_ (mL/min/mL) with a single-tissue compartment model and an automatically captured cardiac image-derived input function. Parametric perfusion images were automatically calculated using the basis-function method with initial voxel-wise delay estimation and a leading-edge approach. Subsequently, perfusion of volumes-of-interest (VOI) can be directly extracted from the parametric images. We used a [^18^F]PSMA-1007 SUV 4 fixed threshold for tumour delineation and transferred these VOIs to the perfusion map.

**Results:**

For 8 primary tumours, 64 lymph node metastases, and 85 bone metastases, median tumour perfusion were 0.19 (0.15–0.27) mL/min/mL, 0.16 (0.13–0.27) mL/min/mL, and 0.26 (0.21–0.39), respectively. The correlation between calculated perfusion from time-activity-curves and parametric images was excellent (*r* = 0.99, *p* < 0.0001).

**Conclusion:**

LAFOV PET imaging using [^15^O]H_2_O enables truly quantitative parametric images of whole-body tumour perfusion, a potential biomarker for guiding personalized treatment and monitoring treatment response.

**Supplementary Information:**

The online version contains supplementary material available at 10.1007/s00259-024-06799-3.

## Introduction

Tumour perfusion is a nutrient-agnostic biomarker for cancer metabolic rate [1, 2]. Tumour perfusion has been measured for characterization of prostate cancer (PCa) [3–9] lung- [10, 11], colorectal- [11, 12], breast- [13–16], brain- [17], and head and neck cancer [18–20].

However, imaging of fast, dynamic processes like perfusion is limited to the field-of-view of the PET scanner, making simultaneous imaging of primary tumours and distant metastases difficult. Besides, kinetic analysis requires an arterial input function, which can be obtained by continuous arterial blood sampling or from a dynamic scan over the left ventricle or a large vessel. To overcome this challenge, either multiple [^15^O]H_2_O PET scans or the use of a long axial field-of-view (LAFOV) PET scanner is required [21].

Consequently, previous studies on tumour perfusion in PCa are mostly restricted to the pelvic region and hence, only little knowledge of the perfusion of PCa metastases exist [3–9]. However, as LAFOV PET scanners are becoming increasingly available, offering superior sensitivity and the possibility to perform dynamic whole-body PET scans, perfusion imaging of the total metastatic burden is possible. In this study, we aim to show how quantitative parametric images of perfusion throughout the body can be created and used to characterize whole-body tumour perfusion in metastatic PCa.

## Materials and methods

### Patient population

Ten patients with metastatic PCa, referred for a clinical [^18^F]PSMA-1007 PET/CT at the Department of Nuclear Medicine and PET-Centre, Aarhus University Hospital were recruited for the study. Two patients were scanned as primary staging and were hence treatment naïve, while eight patients were heavily pretreated patients with castration resistant PCa, scanned at either baseline or as interim scan during [^177^Lu]Lu-PSMA I&T therapy.

The study was approved by the institutional review board (Central Denmark Region Committees on Health Research Ethics (1-10-72-156-22)), and all subjects signed a written informed consent.

### Imaging

All scans were performed on a Siemens Biograph Vision Quadra LAFOV PET/CT scanner (Siemens, Erlangen, Germany). A 400 MBq [^15^O]H_2_O bolus was injected at scan start using a MedRad Contrast Infusion pump (10 mL, 1 mL/s, and 30 mL saline flush), followed by 5-minute dynamic PET acquisition (1 × 10s, 10 × 5s, 6 × 10s, 2 × 15s, 3 × 20s, 3 × 30s). Subsequently, 2.5 MBq/kg [^18^F]PSMA-1007 was injected, and PET acquisition from 60 to 70 min post-injection were used for reconstruction of SUV images.

Images were reconstructed using ordered subset expectation maximum reconstruction algorithm with time-of-flight and point-spread-function and a 3 mm 3D gaussian post-filter. The voxel-size was 3 × 3 × 3 mm. A low-dose CT was used for attenuation correction.

### Image analysis

Tumour volumes-of-interest (VOIs) for both primary prostate lesion and metastatic bone- and lymph node lesions were semi-automatically delineated using a SUV 4 fixed threshold on the [^18^F]PSMA-1007 images, which has shown the best correlation with CT reference volumes for lymph node metastases [22]. Tumour VOIs were drawn using Hermes Affinity Viewer version 3.0.1 (Hermes Medical Solutions, Stockholm, Sweden). Tumour VOIs less than 5 mm in diameter were excluded from analysis. The tumour VOIs were then transferred to the [^15^O]H_2_O PET images, and time-activity-curves were extracted.

**Fig. 1:**
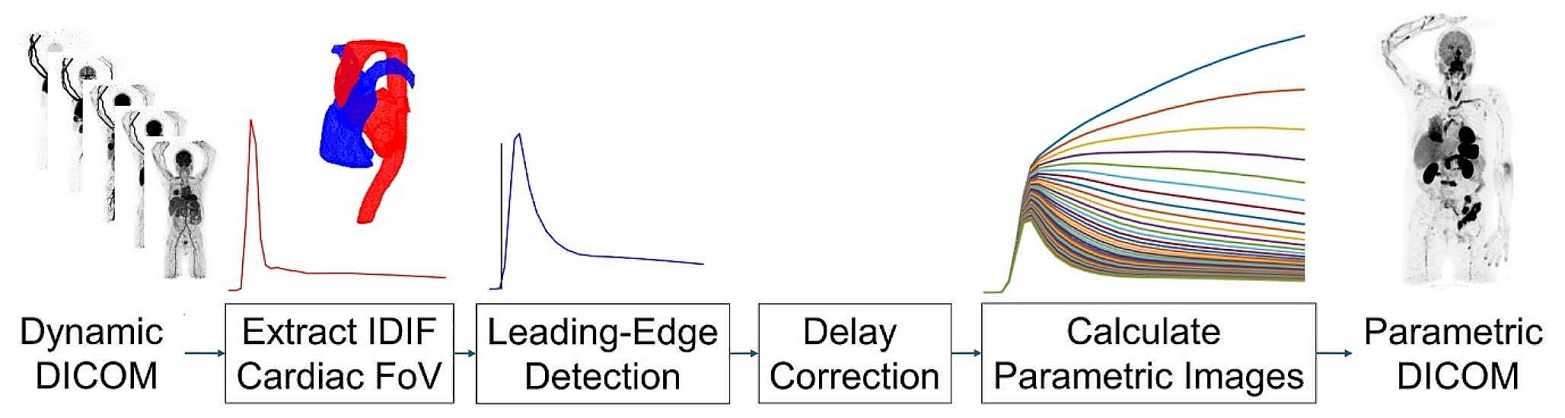
Schematic overview of the processes involved in creation of parametric images

[^15^O]H_2_O K_1_ (mL/min/mL) was used as the metric of perfusion and was calculated using a single-tissue compartment model with arterial and venous blood volumes. All VOI-based kinetic analyses, parametric image calculations, and blood input function extractions were performed using the aQuant Research Package (MedTrace, Hørsholm, Denmark). In short, the cardiac image-derived input functions were extracted automatically from the LAFOV dynamic series by cropping to the lung field-of-field followed by cluster analysis to identify venous and arterial clusters (Fig. 1) [23]. The same blood input functions were used to for both VOI-based analysis and parametric image calculation. VOI-based analysis was performed with delay as a free parameter. Whole-body parametric perfusion images were calculated using the basis-function method [23]. To improve robustness, delay correction was performed prior to parametric image calculation by voxel-wise estimation of delay and a leading-edge approach [24] and, subsequently, correcting each voxel for the delay to yield a delay-free dynamic image series (Fig. 1) (MATLAB, MathWorks, Natick, MA) [25].

### Statistical analysis

Data were tested for normality using QQ-plots and histograms. Data are reported as median with interquartile range (IQR). Non-normally distributed data were log-transformed in parametric analysis. Pearson’s correlation (r) was used for correlation analysis. P values < 0.05 were considered statistically significant.

Statistical analysis was performed in MATLAB.

## Results

**Fig. 2:**
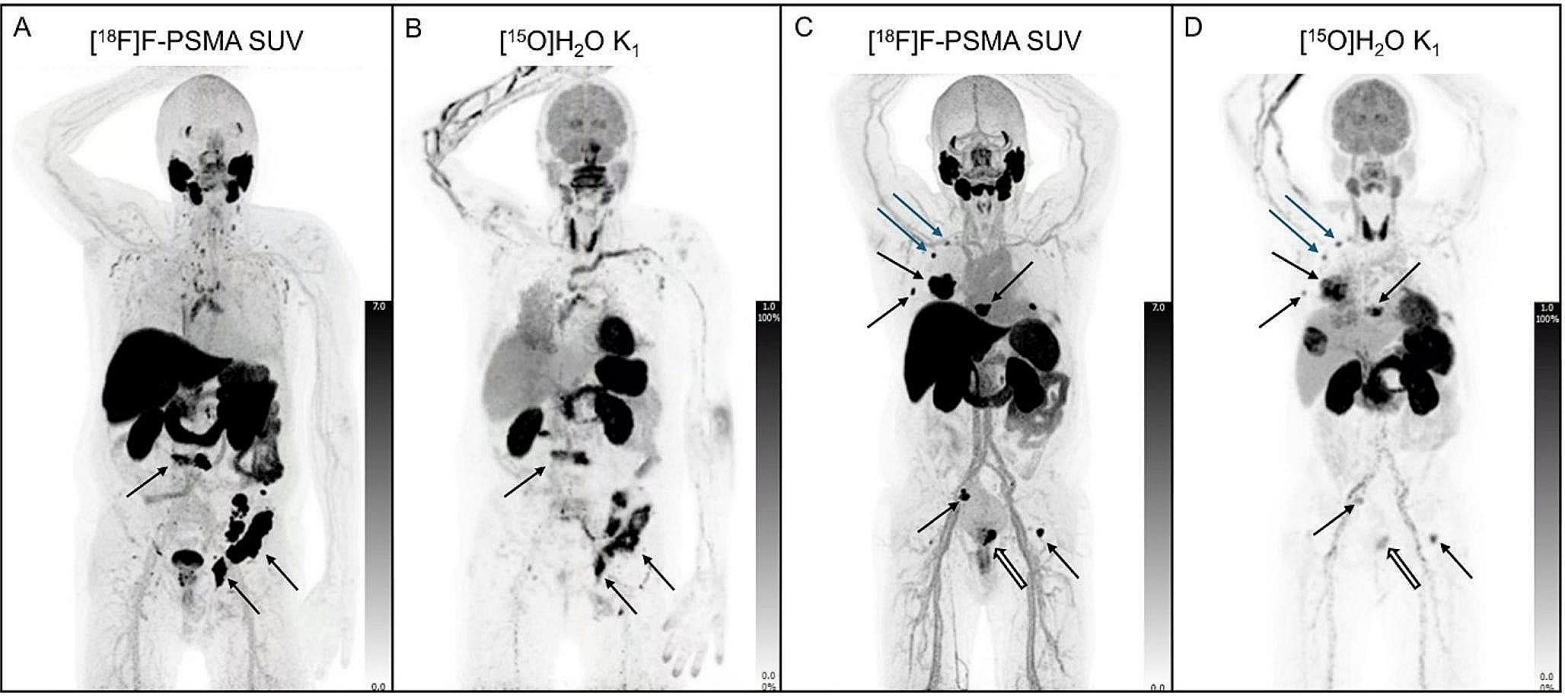
Maximum intensity projections (MIP) of [^18^F]PSMA-1007 SUV images (**A** and **C**) and [^15^O]H_2_O K_1_ (mL/min/mL) (**B** and **D**). The images of the first patient (**A** and **B**) shows highly increased perfusion in pelvic and vertebral bone metastases (black arrows). The second patient (**C** and **D**) has both a primary tumour (white arrow), expansive bone metastases (black arrows) and two small lymph node metastases (blue arrows) with increased perfusion

**Fig. 3:**
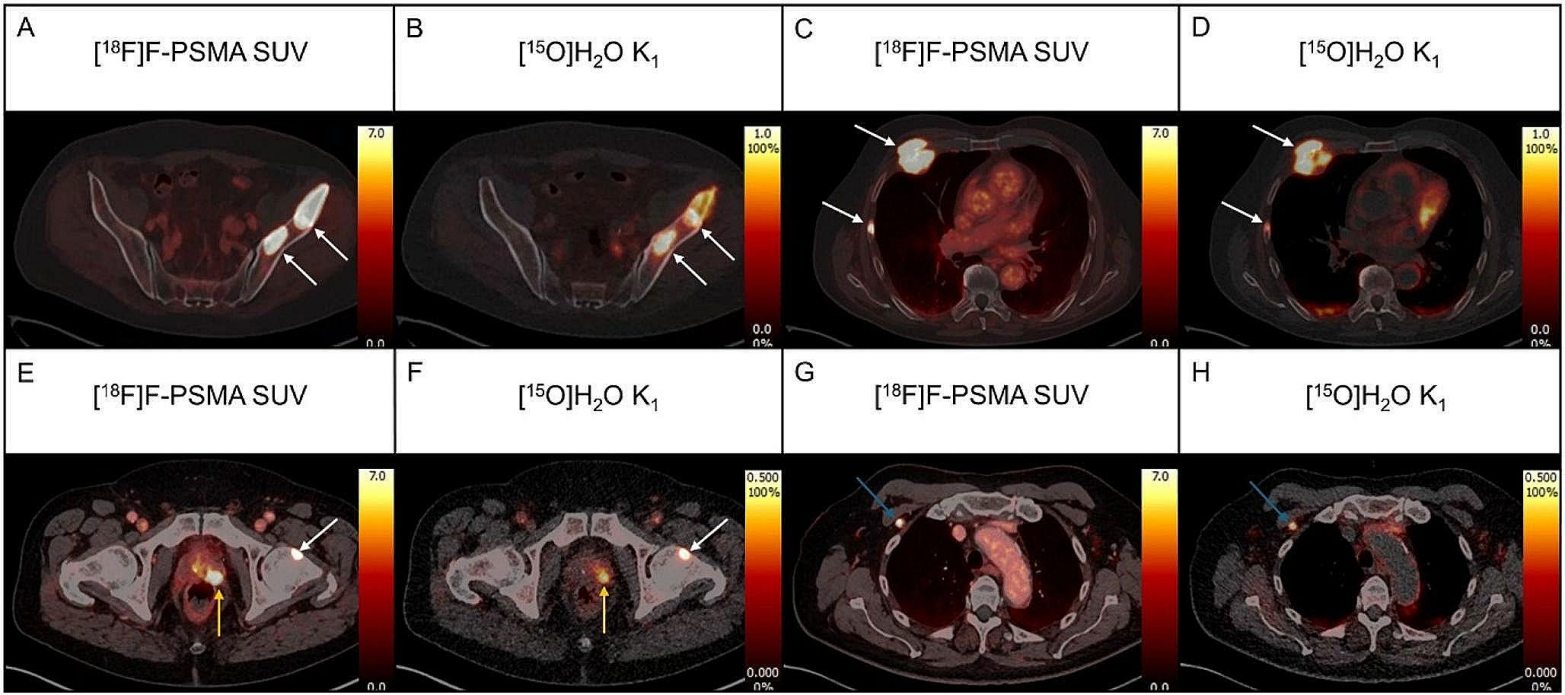
Transaxial fused [^18^F]PSMA-1007 SUV PET/CT images for tumour location (**A**, **C**, **E**, and **G**, arrows) and parametric trans axial fused [^15^O]H_2_O K_1_ images (mL/min/mL) (**B**, **D**, **F**, and **H**) showing highly increased perfusion in bone metastases (**B**, **D** and **F**, white arrows), primary tumour (**F**, orange arrow) and lymph node metastasis (**H**, blue arrow)

Examples of whole-body parametric perfusion images are shown in Figs. 2 and 3, demonstrating both primary tumours and metastases with increased perfusion.

**Fig. 4:**
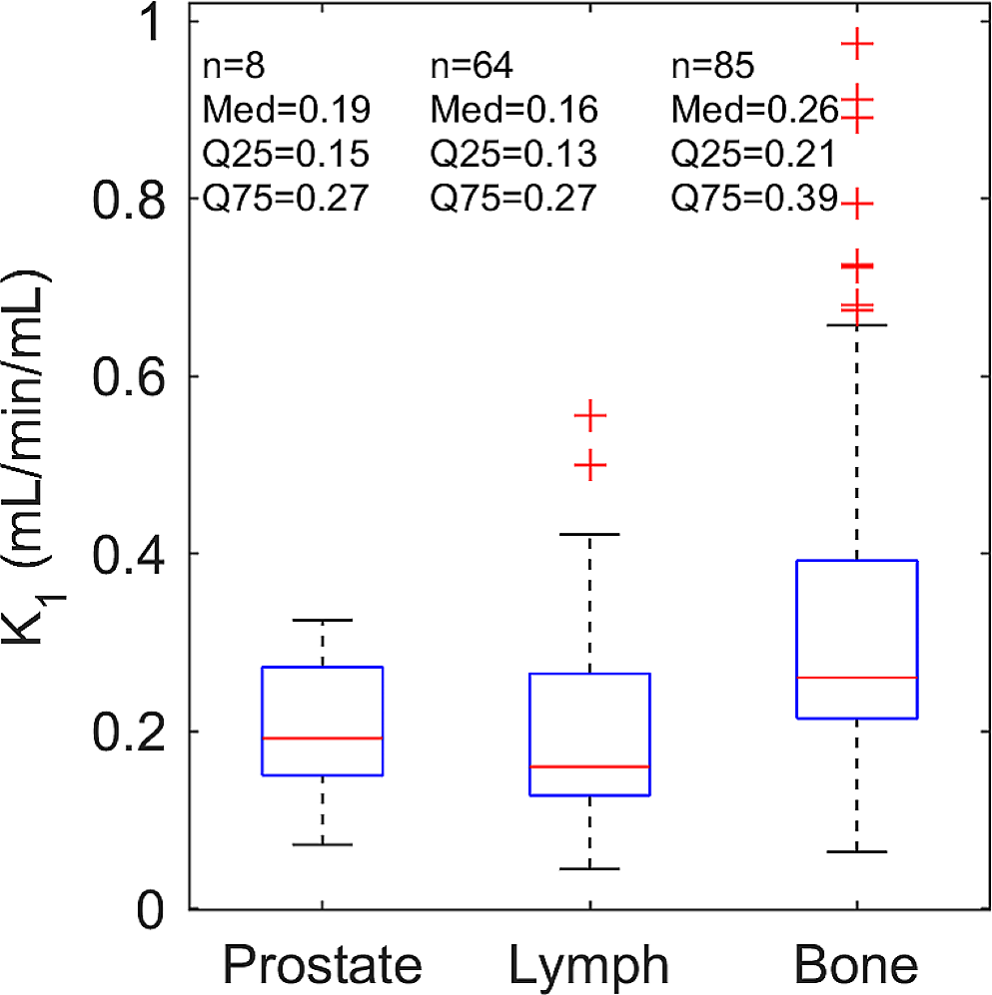
Boxplot of median [^15^O]H_2_O K_1_ perfusion for primary prostate tumours (*n* = 8), lymph node metastases (*n* = 64), and bone metastases (*n* = 85), respectively. Median and interquartile range are listed for each category

Eight primary tumours, 64 lymph node metastases, and 85 bone metastases were defined. Median tumour perfusion for primary tumours, lymph node metastases, and bone metastases were 0.19 mL/min/mL IQR (0.15–0.27 mL/min/mL), 0.16 mL/min/mL, IQR (0.13–0.27 mL/min/mL), and 0.26 mL/min/mL IQR (0.21–0.39 mL/min/mL), respectively (Fig. 4).

The distribution volume (V_d_) of primary tumours, lymph node metastases, and bone metastases were 0.86 IQR (0.84–0.88), 0.39, IQR (0.31–0.48), and 0.55 IQR (0.45–0.61), respectively.

Only a single liver metastasis was observed in the included patients, with tumour perfusion above surrounding liver perfusion. One patient had a few very small lung metastases, which were too small for proper analysis. Due to the small sample size, the quantitative data from these are not reported or included in analysis.

**Fig. 5:**
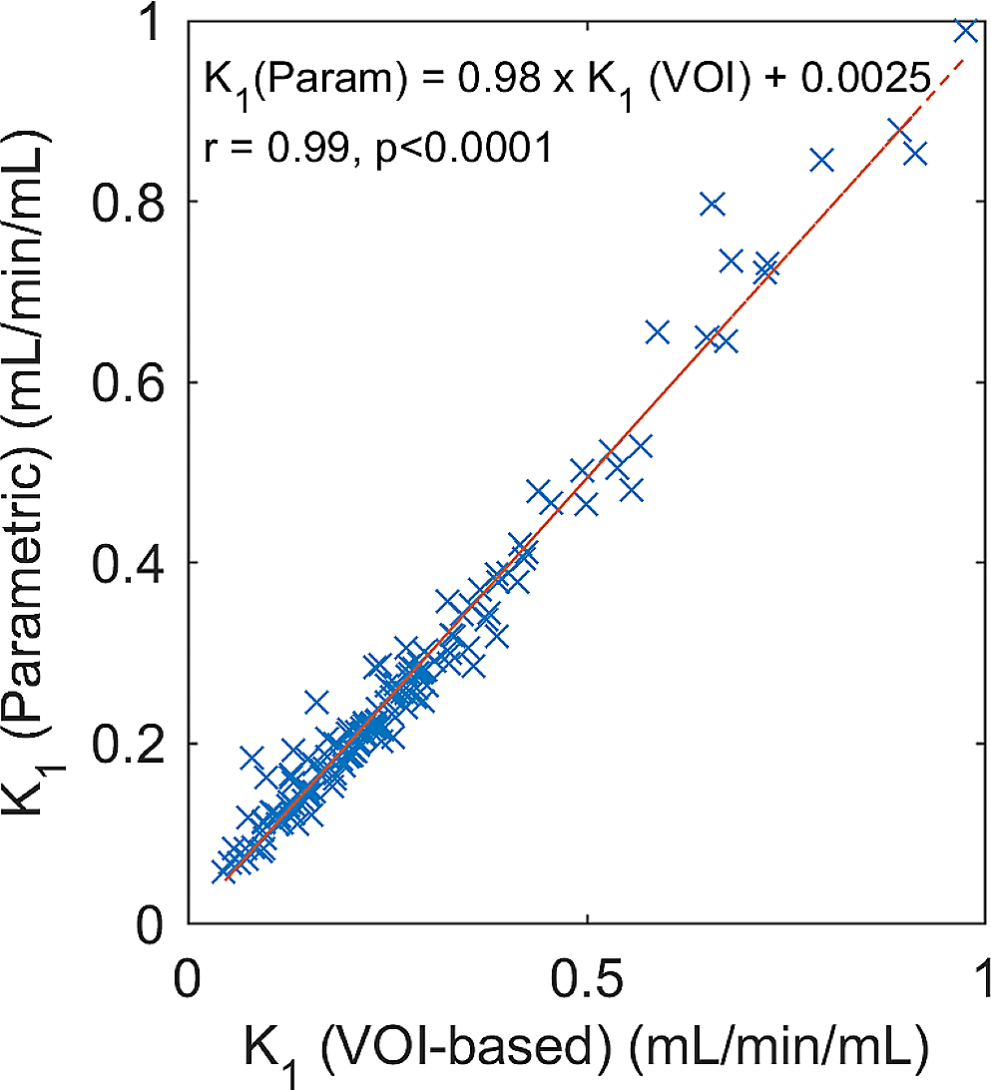
[^15^O]H_2_O K_1_ derived from the parametric images plotted against VOI-based [^15^O]H_2_O K_1_ values reveals an excellent agreement between standard VOI-based methods using delay as a model parameter and parametric images produced using the basis-function method with initial delay-estimation using a leading-edge approach (*r* = 0.99, *p* < 0.0001), suggesting that the parametric images are truly quantitative

In Fig. 5, the mean perfusion derived from parametric [^15^O]H_2_O K_1_ images are plotted against the calculated [^15^O]H_2_O K_1_ from time-activity-curves on dynamic images for each VOI. There was an excellent correlation (*r* = 0.99, *p* < 0.0001) between the mean perfusion values showing that the parametric images are truly quantitative of tumour perfusion.

## Discussion

In the present study we have shown that it is possible to produce whole-body parametric tumour perfusion images, using a LAFOV PET scanner, and that the parametric images calculated using the basis-function method with prior delay correction are truly quantitative. With these tools it is possible to characterize whole-body tumour perfusion in all stages of metastatic prostate cancer and in other metastatic cancers.

The whole-body parametric perfusion images enables both quantitative and qualitative assessment of the patient’s metastatic disease burden, which makes repeated analysis far more feasible both for research and in a clinical setting. To measure very low perfusion levels, as in resting muscle tissue, it may be beneficial to utilize an autoradiographic approach in combination with prior delay correction to parametric image calculation [25].

There is a substantial variation in the perfusion of both primary tumours and metastases. The bone metastases with particularly high perfusion tend to generally represent the largest metastatic deposits. While tumour biology is known to be heterogenous, the heterogeneity might be further enhanced in the heavily pretreated patients, where metastatic tumour deposits might belong to different cancer clones. In addition to biological variation, the individual lesions may contain mixed viable and non-viable tumour tissue within the VOIs and even within each voxel. This fraction of perfused tissue is reflected in the V_d_ of [^15^O]H_2_O [26]. In the current study, V_d_ is largest in the primary tumours, lowest in the small lymph node metastases, whereas bone metastases appear heterogenous with a wide range of V_d_ values.

Using a SUV 4 fixed threshold on the [^18^F]PSMA-1007 images for tumour delineation is validated only on lymph node metastases [22] and hence it could be less suitable for other tumour sites. The whole-body parametric perfusion images allow free choice of tumour delineation method.

Previous studies have shown a correlation between tumour perfusion and primary tumour aggressiveness [5–8], however this topic is not meaningful to assess in the present cohort, as for most patients the relevant biopsies lie several treatments back and are hence no longer representative of current disease aggressiveness.

Whether the described possibility to create whole-body parametric perfusion images provides valuable clinical information needs to be enlightened in future prospective studies across various cancer forms. In PCa the tumour perfusion could be of relevance both in the hormone sensitive and castration resistant phase and should be compared to PSMA-derived metrics. For instance, it is relevant to study whether the pretreatment tumour perfusion is predictive of response to various treatments and whether the changes in tumour perfusion after start of treatment is predictive of long-term treatment effect. Similarly, given that perfusion in all organs is measured simultaneously, it would be of interest to study the systemic impact of tumour aggressiveness, drug effects and co-morbidities.

The presented method is limited by the use of a single kinetic model for all tissues. For some organs more advanced modelling is needed (e.g. lungs and liver), which means the parametric image must integrate several organ specific models [21]. In the current study, lung and liver metastasis were only present in a single patient and were excluded from the analysis. Another limitation could be inflammation in or around lesions, which leads to increased perfusion.

## Conclusion

Using LAFOV [^15^O]H_2_O PET imaging it is possible to create quantitative parametric images of whole-body tumour perfusion, which could be a potential biomarker for guiding personalized treatment and monitoring response to various treatments.

## Electronic supplementary material

Below is the link to the electronic supplementary material.

Supplementary Material 1

Supplementary Material 2

## Data Availability

The datasets used in the current study are available from the corresponding author on reasonable request.
