# Supplementary figures and images for: Whole-body parametric mapping of tumour perfusion in metastatic prostate cancer using long axial field-of-view [15O]H2O PET
Source: Eur J Nucl Med Mol Imaging. 2024 Jun 28;51(13):4134–40. doi: 10.1007/s00259-024-06799-3 (PMC11527927; doi:10.1007/s00259-024-06799-3)

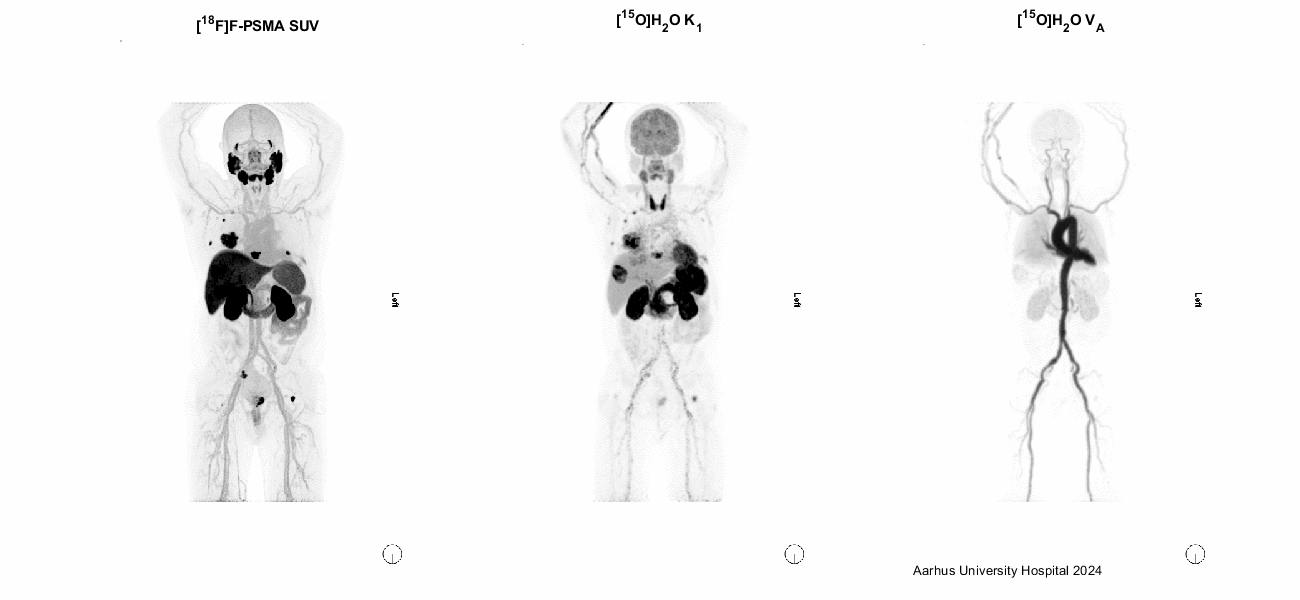

Supplement: Supplementary file 1 — Supplementary Material 1 [file 259_2024_6799_MOESM1_ESM.gif]

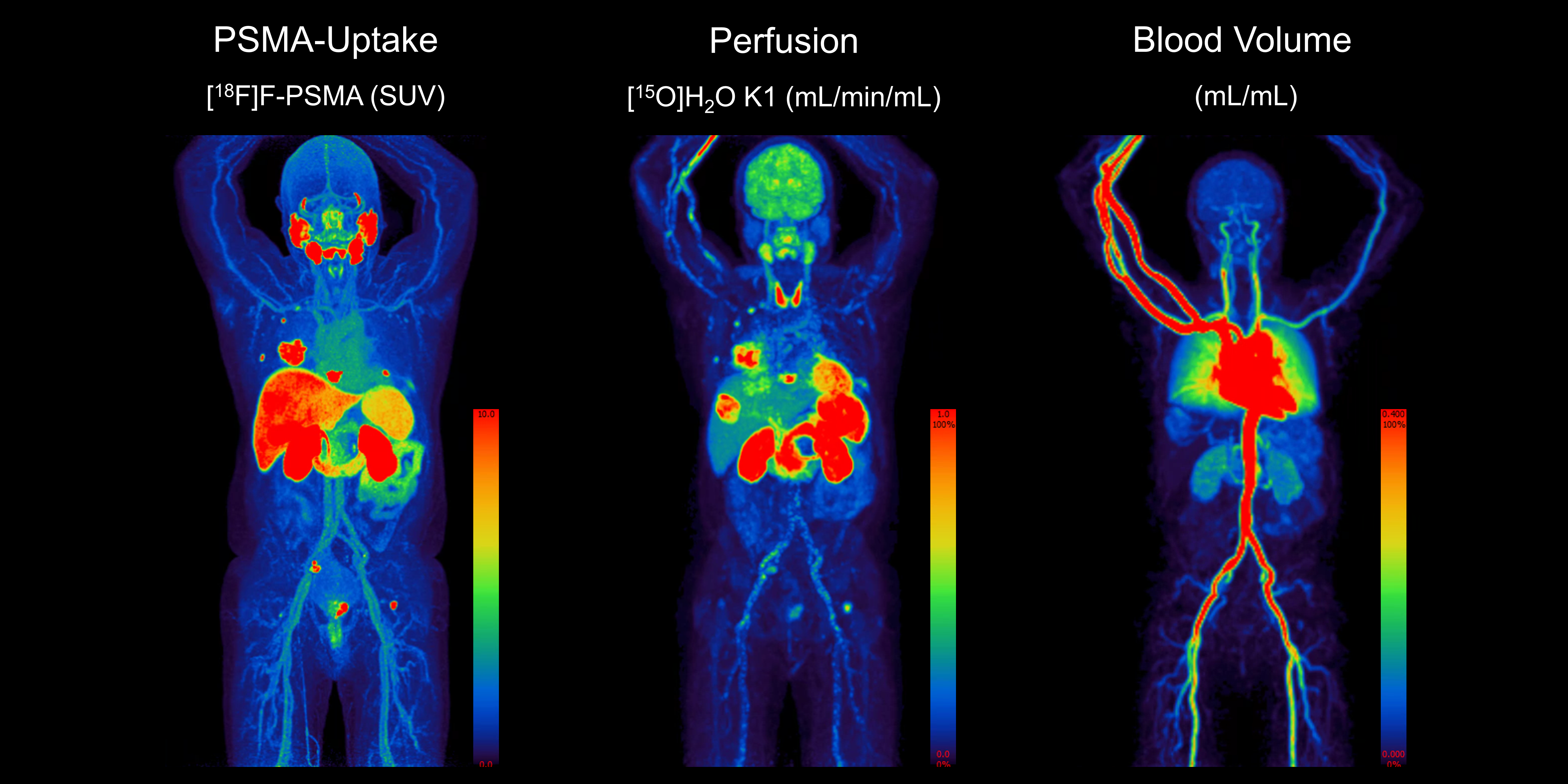

Supplement: Supplementary file 2 — Supplementary Material 2 [file 259_2024_6799_MOESM2_ESM.png]
